# White Light Orchestrates Mycoparasitic and Infection Activities by Regulating Expression of Effectors in *Trichothecium roseum*

**DOI:** 10.1002/fsn3.70396

**Published:** 2025-06-28

**Authors:** Mo Zhu, Fuhai Zhang, Zongbo Qiu, Sujing Zhao, Shiqiang Gao

**Affiliations:** ^1^ College of Life Sciences Henan Normal University Xinxiang P.R. China; ^2^ Henan Center for Outstanding Overseas Scientists Henan Normal University Xinxiang P.R. China; ^3^ Xinxiang Key Laboratory of Plant Stress Biology Xinxiang P.R. China; ^4^ Henan International Joint Laboratory of Agricultural Microbial Ecology and Technology Henan Normal University Xinxiang P.R. China; ^5^ Department of Neurophysiology, Institute for Physiology University of Würzburg Würzburg Germany; ^6^ The Observation and Research Field Station of Taihang Mountain Forest Ecosystems of Henan Province Xinxiang Henan China

**Keywords:** effector, infection, mycoparasitism, *Trichothecium roseum*, wheat powdery mildew fungus

## Abstract

The fungal developmental processes are orchestrated by white light. Despite the genome assembly of *Trichothecium roseum* being available, the underlying molecular mechanisms of the white light‐mediated developments of *T. roseum* remain obscure. It was found that white light impaired mycoparasitic activities against the wheat powdery mildew fungus and infection processes on tomato fruits in *T. roseum* . In vitro and in vivo, white light significantly impaired colony expansion and dramatically increased conidiation of *T. roseum* . RNA‐seq analysis of *T. roseum* conidia was profiled to illustrate the light‐mediated expression of genes. A total of 153 and 666 differentially expressed genes were identified between conidia treated with or without white light at 48‐ and 96‐h post inoculation (hpi). Among genome‐wide identified effectors, 8 and 36 effectors were differentially regulated by white light at 48 and 96 hpi, respectively. The core effectors, Tro004101, Tro006854, Tro008316, and Tro004104 were commonly downregulated by white light. Notably, white light regulated gene expression in key metabolic pathways including tryptophan metabolism (3 genes) and tyrosine metabolism (5 genes), as well as the HOG‐MAPK signaling cascade. These results demonstrated that white light‐compromised *T. roseum* mycoparasitic and infection activities might be achieved by regulating specific effector expression and differentially modulating metabolism and HOG‐MAPK pathways. The genes detected by our transcriptome analysis may be crucial for mycoparasitism and infection by *T. roseum* and thus serve as targets for future functional analysis. Our findings provide new insights into the white light‐orchestrated developments of an important agricultural and economical fungus and will potentially support efforts for the study of fungal effectors.

## Graphical abstract

White light‐mediated mycoparasitic and infection activities of *Trichothecium roseum.*

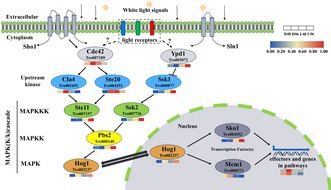

## 1 Introduction

Light controls diverse physio‐morphological responses in fungi by orchestrating developmental and metabolic processes, such as infection, mycoparasitic activities, sporulation, sexual fruiting body formation, pigmentation, and secondary metabolite biosynthesis (Yu and Fischer 2019; Bayram and Bayram 2023). Fungi utilize photoreceptors to sense near‐ultraviolet, blue, green, red, and far‐red light, facilitating environmental adaptation (Corrochano 2019). Fungi sense short, middle, and long wavelengths of light with three types of photoreceptors, including blue light receptors (white collars, envoys, cryptochromes, and photolyases), green light receptors (opsins), and red light receptors (phytochromes) (Schumacher 2017). Although the effects of light on the mycoparasitic activities of fungi were repeatedly illustrated, the underlying molecular mechanisms remained obscure (Karlsson et al. 2017; Moreno‐Ruiz et al. 2020; Speckbacher et al. 2020; Sun et al. 2024).

The *Trichothecium* genus consists of approximately 80 species, which mostly are plant pathogenic fungi (http://www.mycobank.org/). *Trichothecium roseum* is widely known as a primary fungal pathogen causing a rot disease that infests various vegetables, fruits, and crops (Barnett and Hunter 1972), including cucumber, tomato, apple, orange, mango, Hami melon, strawberry, tea, peppers, and maize (Yang et al. 2003; Kasuyama and Tanina 2007; Dal Bello 2008; Kwon et al. 2010; Inácio et al. 2011; Hamid et al. 2014; Lin et al. 2016; Xue et al. 2016; Li et al. 2022; Zhu et al. 2023; Lei et al. 2024). Although *T. roseum* is regarded as the causal agent of pink rot on agriculturally and economically important crops, it is also a biocontrol agent (BCA) against multiple plant diseases, including fungi and insects, *Pauropsylla buxtoni, Phakopsora pachyrhizi, Sclerotinia sclerotiorum, Rhizoctonia solani, Penicillium digitatum*, and *Blumeria graminis* f. sp. *tritici* (Freeman and Morrison 1949; Huang and Kokko 1993; Tesfagiorgis and Laing 2010; Zhang et al. 2010; Kumar and Jha 2012; Wei et al. 2018; Batta 2020; Zhu, Duan, Cai, Zhang, et al. 2022). Despite the availability of a high‐quality genome assembly for *T. roseum* (Zhu et al. 2023), the molecular mechanisms underlying its dual ecological roles in host infection and mycoparasitic activities remain poorly characterized.

*T. roseum* exhibits dual ecological associations with different host substrates, for example, *T. roseum‐*plant hosts and *T. roseum‐*fungi systems (Dai et al. 2024; Zhang et al. 2024; Zhao et al. 2024). During *T. roseum‐*plant host interactions, it acts as a pathogen to invade plant leaves and fruits. However, in the *T. roseum‐*fungus interactions, it is regarded as a mycoparasite and/or BCA. Nevertheless, to successfully establish colonization, *T. roseum* needs to utilize various strategies for infection and hyperparasitism. Effectors are known to suppress the plant host resistance response to complete colonization and to enhance the biocontrol efficiency of fungi against plant diseases (Laur et al. 2018; Santhanam et al. 2023; Seong and Krasileva 2023). However, little is known about the mechanisms of highly complex *T. roseum‐*host interactions and which specific effectors are involved in the infection and mycoparasitic activities.

The main objectives of this study were to (1) functionally characterize the impacts of white light on the infection and mycoparasitic activities of *T. roseum* and (2) illustrate the molecular mechanisms of white light‐mediated gene expression, especially the regulation of effectors and genes in pathways. The results of this study enhance our understanding of the infection and mycoparasitic biology and effectors of *T. roseum* . The findings provide a foundation for investigating the molecular mechanisms of mycoparasitism and infection in other agriculturally and economically relevant plant fungal pathogens and BCAs.

## 2 Materials and Methods

### 2.1 Plant and Fungal Material

A previously isolated *T. roseum* (strain ZM‐Tr2021) was propagated on potato dextrose agar (PDA) in darkness (6 h/8 h light/dark at 20°C) (Zhu, Duan, Cai, Zhang, et al. 2022). *B. graminis* f. sp. *tritici* (Bgt) (isolate Bgtzm2022) was used as the fungal material for *T. roseum* mycoparasitic experiments and was propagated on wheat (cv. Aikang 58) leaves in the growth chamber (temperature, 20°C; light/dark, 16 h/8 h; humidity, 70%) (Zhu, Duan, Cai, Li, and Qiu 2022; Zhu, Duan, Cai, Zhang, et al. 2022). One day before Bgt inoculation, the mildew‐infected leaves were shaken to obtain fresh conidia.

### 2.2 In Vivo Mycoparasitic and Infection Activities of *T. roseum*

To determine *T. roseum* mycoparasitic activities, conidia of Bgt were inoculated onto healthy wheat leaves. At 6 days post inoculation (dpi), mildew‐infected plants were inoculated with *T. roseum* spore suspension (1 × 10^6^ spores mL^−1^) and kept in a growth chamber with or without white light (temperature, 25°C; white light, 100 μmol m^−2^ s^−1^; humidity, 70%). Bgt‐inoculated plants treated with water were served as controls. At 2, 4, and 6 dpi, the wheat leaves were collected for macro‐ and microscopic analysis with a staining method (Zhu et al. 2017). The biomass changes of Bgt and *T. roseum* at 2, 4, and 6 dpi were determined using a previous method with three biological replicates (Zhu, Duan, Cai, Zhang, et al. 2022).

To assay *T. roseum* infection activities, tomato (cv. Zhengfan) fruit was wounded and inoculated with 1 μL *T. roseum* spore suspension (1 × 10^6^ spores mL^−1^). The inoculated fruit was kept in a growth chamber with or without white light (temperature, 25°C; white light, 100 μmol m^−2^ s^−1^; humidity, 80%). The colony sizes of *T. roseum* were measured from 2 to 7 dpi with three biological replicates.

### 2.3 In Vitro Colony Expansion and Conidiation of *T. roseum*

To analyze the developments of *T. roseum* , the colony sizes and spore production were quantitatively measured. Pieces of PDA (1 mm^2^) with *T. roseum* were placed onto the center of petri dishes with PDA. The inoculated petri dishes were transferred into growth chambers with or without white light (temperature, 25°C; white light, 100 μmol m^−2^ s^−1^). To compare the spore production, 20 μL *T. roseum* spore suspension (1 × 10^6^ spores mL^−1^) was homogenously spread onto PDA in petri dishes, and the dishes were placed into growth chambers with or without white light (temperature, 25°C; white light, 100 μmol m^−2^ s^−1^). The colony sizes and spore production were measured from 2 to 7 dpi with three biological replicates.

### 2.4 RNA Extraction, Library Construction, and Sequencing

The total RNA of *T. roseum* conidia on PDA with or without white light treatment was extracted at 48 and 96 hpi according to a previously established method (Zhu et al. 2019). Three biological replicates were used for each treatment group. To construct RNA‐seq libraries, the VAHTS Universal V6 RNA‐seq Library Prep Kit was applied according to the manufacturer's instructions. RNA integrity was assessed using the Agilent 2100 Bioanalyzer (Agilent Technologies, Santa Clara, CA, USA). RNA with RIN (RNA integrity number) > 7 was employed for sequencing. 150‐bp sequence length reads were generated by using the DNBSEQ‐T7 platform with paired‐end sequencing. Low‐quality reads were removed from the raw reads using FASTP (Chen et al. 2018) and clean reads were mapped to the reference genome of *T. roseum* ZM‐Tr2021 (https://www.ncbi.nlm.nih.gov/datasets/genome/GCA_022701375.1/) (Zhu et al. 2023) using HISAT2 software (Kim et al. 2015). The fragments per kilobase of transcript per million mapped reads (FPKM) of *T. roseum* each gene were calculated, and the read counts were obtained using HTSeq‐count (Roberts et al. 2011; Anders et al. 2015). Sequence data were deposited in the NCBI Short Read Archive (SRA) with accession number (BioProject ID PRJNA1110597). Transcriptome sequencing and analysis were conducted by OE Biotech Co. Ltd. (Shanghai, China).

### 2.5 Differentially Expressed Genes

The differentially expressed genes (DEGs) were detected using DESeq2 (Love et al. 2014). Genes with a combination of *q*‐value < 0.05 and [log_2_ FC > 1] were regarded as DEGs. Subsequently, according to the hypergeometric distribution, Gene Ontology (GO) (The Gene Ontology Consortium 2018), Kyoto Encyclopedia of Genes and Genomes (KEGG) (Kanehisa et al. 2008) enrichment analyses of the DEGs were performed to screen significantly enriched terms using R (v3.2.0). GO enrichment analysis was used to filter the DEGs relevant to biological processes, cellular components, and molecular functions (Ashburner et al. 2000). For KEGG enrichment analysis, which was used for identifying significantly enriched metabolic or signal transduction pathways in DEGs, FDR ≤ 0.05 was considered as a threshold (Kanehisa and Goto 2000). Venn diagrams and heatmaps were constructed using the TBtools (V2.096) software with the mean value of FPKM from three biological replicates (Chen et al. 2023).

### 2.6 Identification of Effector Encoded by the *T. roseum* Genome

The *T. roseum* effectors were identified according to classical screening conditions for effector proteins in plant pathogenic fungi. Firstly, proteins (length ≤ 300 aa) that contain a signal peptide in the N‐terminal, without transmembrane domain and GPI anchoring site, with at least one disulfide bond were screened. The protein sequences were submitted to SignalP‐6.0 for detection of signal peptides (Teufel et al. 2022). The transmembrane structure was predicted using TMHMM v2.0c (https://services.healthtech.dtu.dk/services/TMHMM‐2.0/). All the remaining sequences were screened by EffectorP‐fungi 3.0 (https://effectorp.csiro.au/) with a threshold of at least 0.6 (Krogh et al. 2001; Sperschneider and Dodds 2021).

### 2.7 Validation of Differentially Expressed Genes by RT‐qPCR

Thirteen genes encoding light receptors and in the HOG‐MAPK pathway were randomly selected for detection of transcription by real‐time quantitative PCR. Specific primers were designed using TBtools (Table S3). RNA was reverse transcribed into cDNA using the M5 Super Plus qPCR RT kit with a gDNA remover (Mei5, Beijing, China). The RT‐qPCR reactions were conducted using the 2 × qPCR SYBR Green Master Mix (Vazyme, Ningjing, China) with a LightCycler 96 real‐time PCR instrument (Roche, Switzerland). The cycling conditions were as follows: a pre‐denaturation at 95°C for 300 s, 45 cycles with each cycle employing a denaturation temperature at 95°C for 10 s, and an annealing/extension temperature at 60°C for 30 s (Zhu et al. 2024). For the melting curve setting, the instrument default was used: 95°C for 10 s, 65°C for 60 s, and 97°C for 1 s. The elongation factor 1 alpha‐encoding gene of *T. roseum* was used as an internal control (Zhu, Duan, Cai, Zhang, et al. 2022). The gene expression levels were calculated using the $2^{-\Delta \Delta C_{T}}$ method (Livak and Schmittgen 2001).

### 2.8 Statistical Analysis

Statistical analyses of gene expression in RT‐qPCR were based on *n* = 3 independent biological experiments, where *n* = 1 represented three technical replicates. Significant differences (*p* < 0.05) between multiple datasets were tested by one‐way ANOVA, followed by a Tukey post hoc test (both significant differences of normality and homogeneity of variance were > 0.05, Shapiro test, and Levene's test). Statistical analyses were performed using IBM SPSS Statistics (version 25.0; IBM Corporation, Armonk, NY, USA).

## 3 Results

### 3.1 White Light Impairs Mycoparasitic Activities of *T. roseum*

**FIGURE 1:**
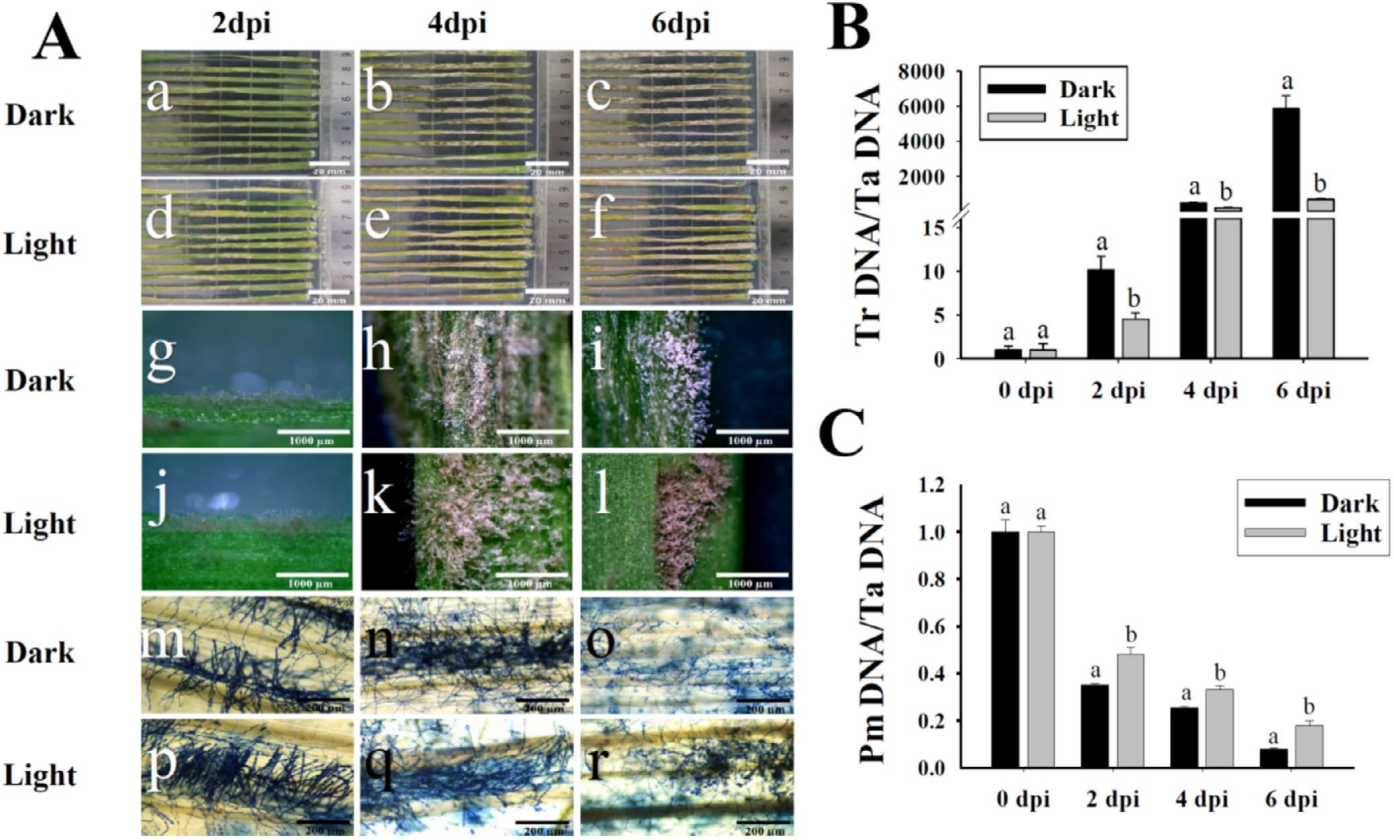
Mycoparasitic activities of *T. roseum* on the wheat powdery mildew fungus *Blumeria graminis* f. sp. *tritici* in darkness and under white light condition. (A) Macroscopic (a–l) and microscopic (m–r) images of *T. roseum* during mycoparasitic processes. (B, C) Biomasses changes of *T. roseum* and powdery mildew (Pm) during the mycoparasitic interactions. The *T. roseum* spore suspension was inoculated onto mildew‐infected wheat leaves and incubated in darkness or under white light conditions for 2, 4, and 6 days. The fungal structures were stained with trypan blue. The biomass ratios were calculated according to powdery mildew fungus (Pm) biomasses/wheat (Ta) biomasses or *T. roseum* (Tr) biomasses/wheat (Ta) biomasses under the conditions of darkness or white light. Significant differences (*p* < 0.05) between datasets were indicated by different letters and were tested by one‐way ANOVA, followed by a Tukey post hoc test.

To analyze the effects of white light on the developments of *T. roseum* , the mycoparasitism of *T. roseum* on the wheat powdery mildew (PM) fungus *B. graminis* f. sp. *tritici* (Bgt) was assayed (Figure 1). Although *T. roseum* was capable of significantly reducing the conidiophores of Bgt on wheat leaves in darkness, the developments and mycoparasitic activities of *T. roseum* were notably impaired under the white light condition. In darkness, massive visible colonies of *T. roseum* formed at 2 dpi, started to hyperparasite on Bgt colonies at 4 dpi, and dramatically inhibited conidiation and conidial distribution of Bgt at 6 dpi (Figure 1a–c,g–i,m–o). However, under white light conditions, only scarce colonies of *T. roseum* formed at 2 dpi, started to form massive colonies on Bgt at 4 dpi, and began to hyperparasite on Bgt colonies at 6 dpi. To quantitatively examine the biomass changes of *T. roseum* and Bgt, the biomasses of these two fungi were measured (Figure 1B,C). The powdery mildew (PM) biomasses were significantly reduced by *T. roseum* mycoparasitism. However, the biomasses of PM in darkness were notably lower than those under white light conditions. Although the *T. roseum* biomasses were dramatically increased during mycoparasitic activities, the biomasses in darkness were significantly higher than those under white light conditions.

### 3.2 White Light Compromises Infection Activities of *T. roseum*

**FIGURE 2:**
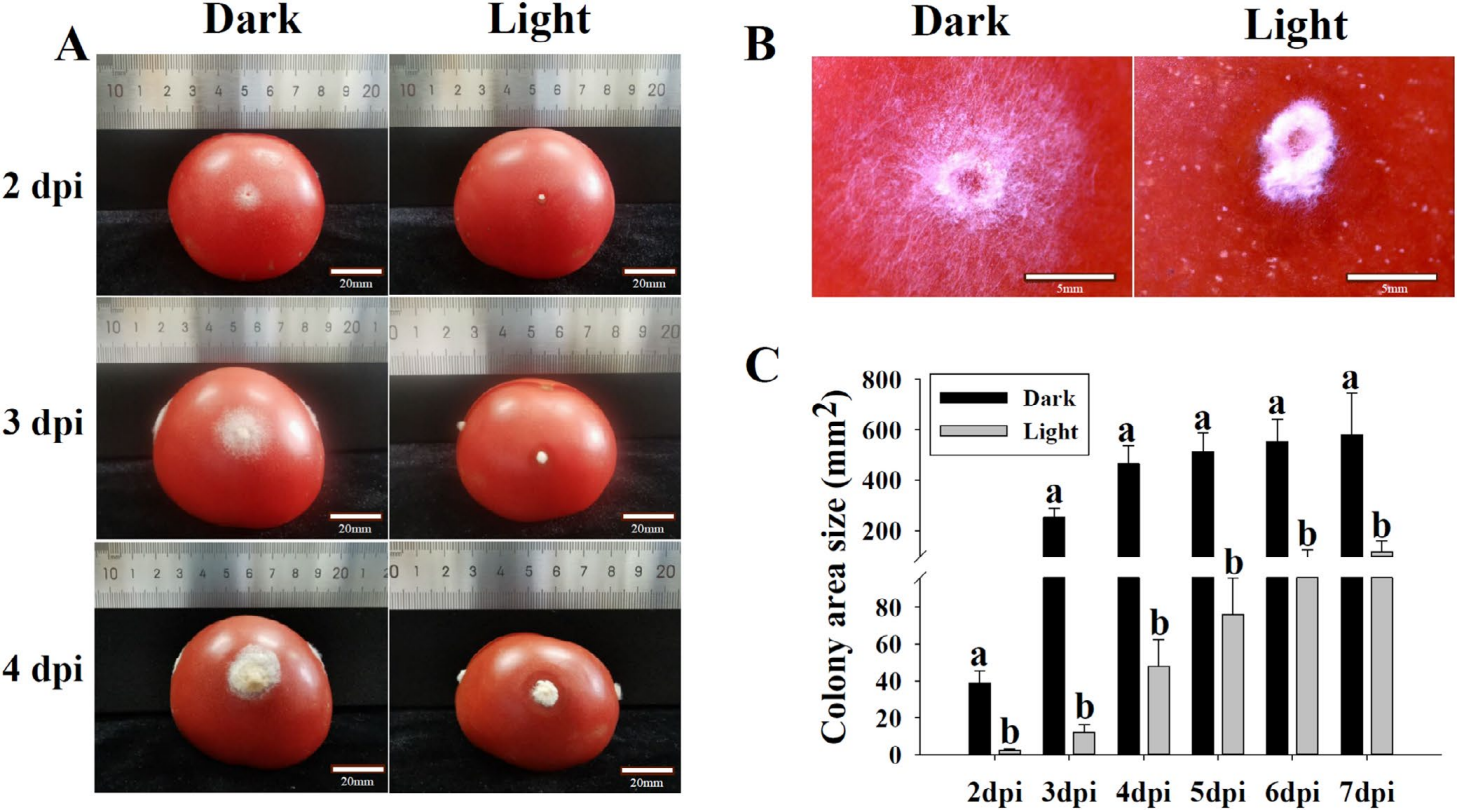
Colony developments of *T. roseum* on tomato fruit in darkness or under white light condition. (A) Macroscopic images of *T. roseum* developments on tomato fruit at 2, 3, and 4 dpi. (B) Comparison of colonies on tomato fruit in darkness or under white light condition at 3 dpi. (C) Colony sizes of *T. roseum* on tomato fruit in darkness or under white light condition. The injured fruit were inoculated with spore suspension of *T. roseum* and incubated in darkness or white light condition for 2–7 days. Bars in (A) were 20 mm and in (B) were 5 mm. Significant differences (*p* < 0.05) between datasets at each time point were indicated by different letters and were tested by one‐way ANOVA, followed by a Tukey post hoc test.

Since *T. roseum* was known to be a pathogen infecting tomato fruit, the effects of white light on infection activities of this fungus were assayed (Figure 2). On tomato fruit, the colonies of *T. roseum* were expanded from injured holes at 2 dpi and produced massive conidia at 3 and 4 dpi in darkness. Under white light conditions, *T. roseum* was not capable of expanding colonies from injured holes of tomato fruit at 2 dpi. Compared to those in darkness, the colony sizes of *T. roseum* under white light conditions were significantly smaller (Figure 2A). At 2 dpi, the conidia were notably formed under white light conditions, whereas there were only little conidia produced in darkness (Figure 2B). To quantitatively compare the developments of *T. roseum* on tomato fruit with or without light treatment, colony sizes were measured. The colony sizes of *T. roseum* under white light conditions were significantly smaller than those in darkness from 2 to 7 dpi (Figure 2C).

### 3.3 In Vitro White Light Triggers Conidiation and Inhibits Colony Expansion in *T. roseum*

**FIGURE 3:**
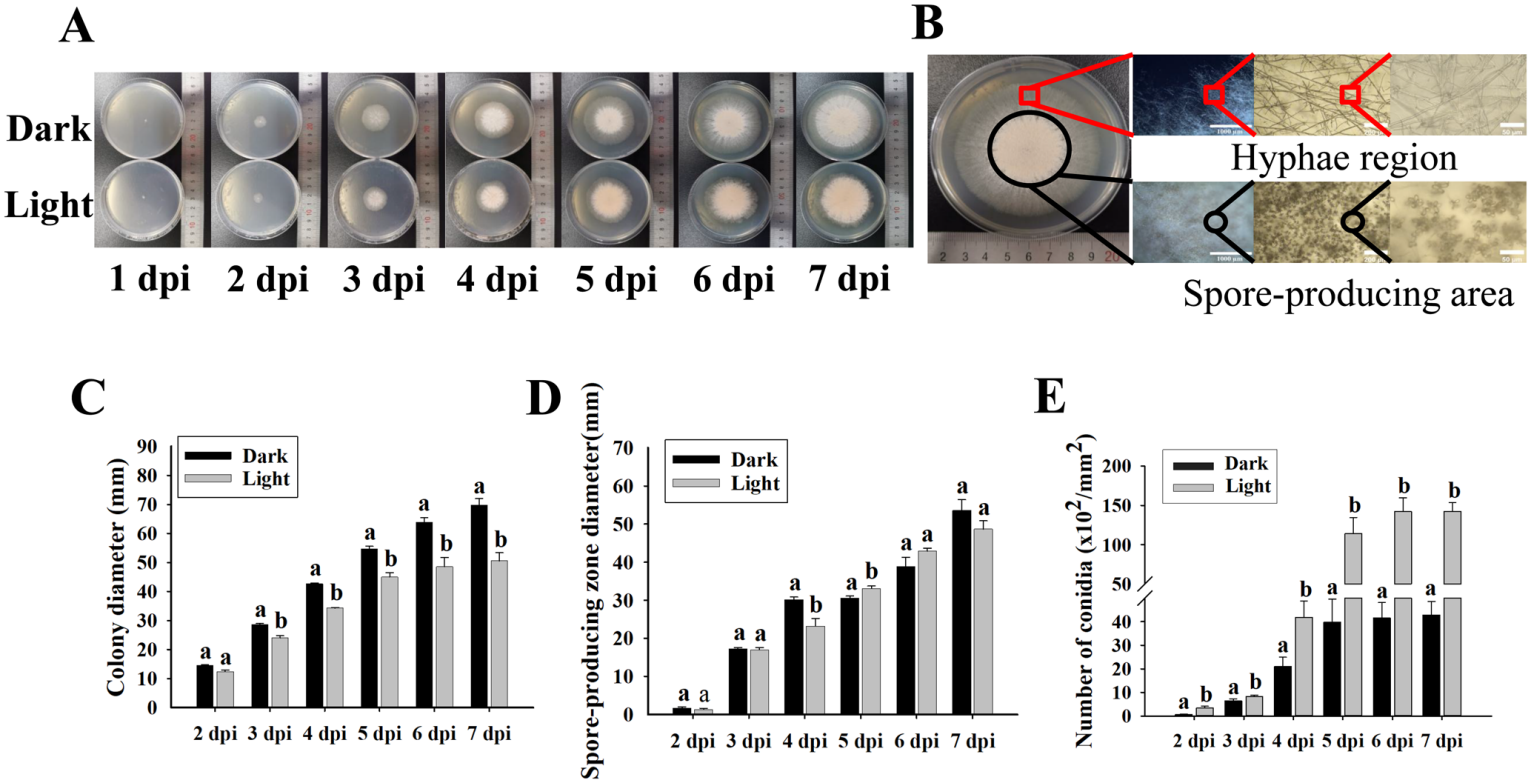
Sizes and produced conidia of *T. roseum* colonies in vitro in darkness or under white light condition. (A) Macroscopy images of *T. roseum* colonies on potato dextrose agar (PDA) from 1 to 7 dpi in darkness or under white light condition. (B) The two distinct hyphae and spore‐producing regions of a *T. roseum* colony. Sizes of *T. roseum* colonies (C) and spore‐producing regions (D). (E) Numbers of produced conidia per colony. The pieces of PDA containing *T. roseum* colonies were placed in the center of the petri dishes with PDA and incubated in darkness or under white light condition for 2–7 days. Significant differences (*p* < 0.05) between datasets were indicated by different letters and were tested by one‐way ANOVA, followed by a Tukey post hoc test.

Since there were two distinct mycelial and spore‐producing regions of *T. roseum* colony (Figure 3A,B), the sizes of the colony and spore production were quantitatively analyzed in vitro. The colony sizes in darkness were significantly higher than those under white light conditions (Figure 3C). Although the sizes of spore‐producing regions were slightly affected, the numbers of produced conidia under white light conditions were significantly higher than those in darkness (Figure 3D,E).

**FIGURE 4:**
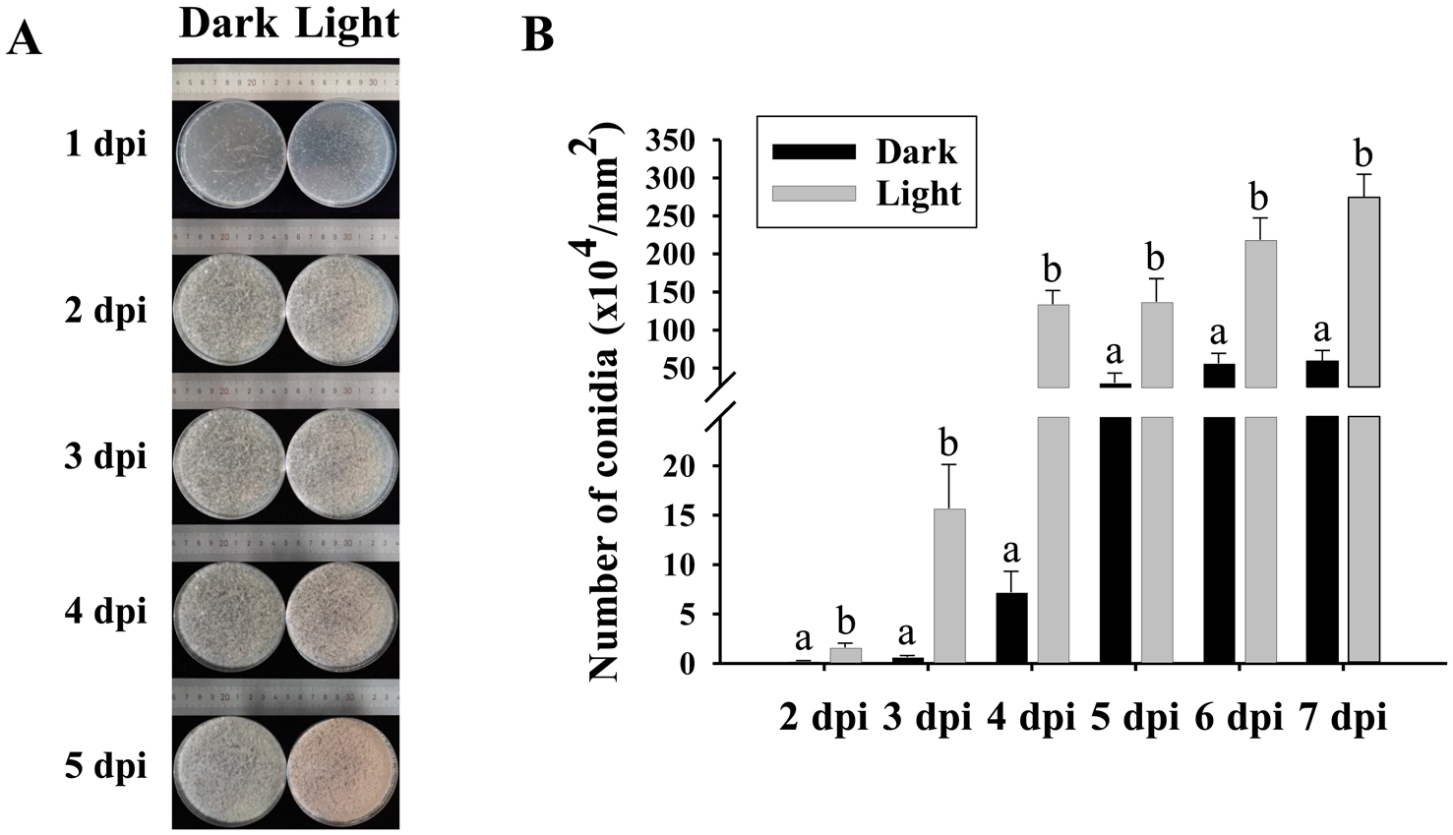
(A) Macroscopic images of colony formation and conidiation in *T. roseum* on PDA from 1 to 5 dpi. The pore suspensions of *T. roseum* were homogenously distributed onto PDA and incubated in darkness or under white light condition. (B) Numbers of produced conidia per petri dish in darkness or under white light condition. Significant differences (*p* < 0.05) between datasets were indicated by different letters and were tested by one‐way ANOVA, followed by a Tukey post hoc test.

To clearly demonstrate the time points of colony formation and conidiation, spore suspensions of *T. roseum* were homogenously distributed onto PDA and the numbers of produced conidia from the fungus in darkness and under white light condition were measured (Figure 4). Colonies were formed at 1 dpi and conidia were produced at 2 dpi (Figure 4A). However, the numbers of produced conidia in darkness were notably lower than those under white light condition from 2 to 7 dpi (Figure 4).

### 3.4 White Light‐Mediated Transcriptome Changes of *T. roseum*

**FIGURE 5:**
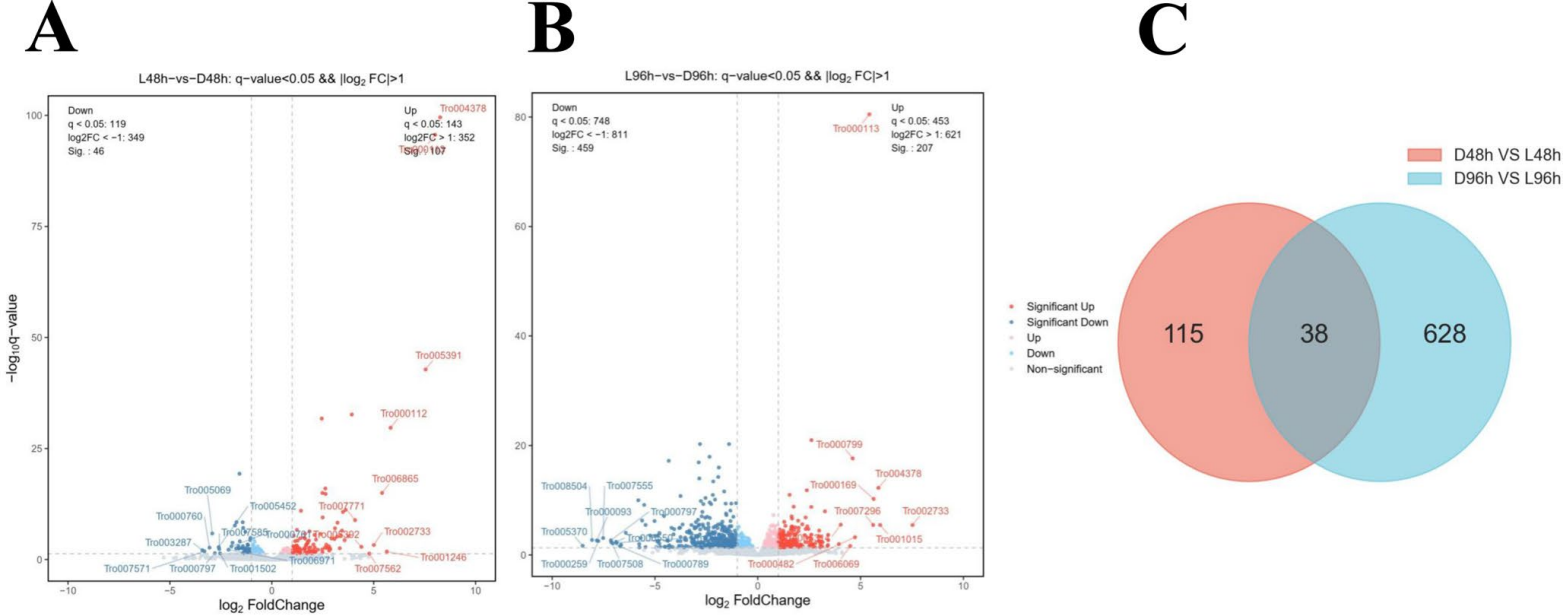
Overview of transcriptome datasets of *T. roseum* in darkness or under white light condition at 48 and 96 hpi. (A, B) Volcano plot of the distribution of DEGs at 48 and 96 hpi. Red dots represent significantly upregulated genes, blue dots represent significantly downregulated genes, and gray dots represent other genes, (C) A Venn diagram showing the numbers of shared or specific DEGs among comparisons.

To study the regulatory mechanisms of white light on *T. roseum* during mycoparasitic and infection activities, the RNA sequencing data of conidia at 0, 48, and 96 h post inoculation (hpi) were analyzed (Figure 5). The FPKM of each gene was calculated based on the length of the gene and the numbers of reads for that gene. A total of 819 DEGs were identified in the two comparison groups. At 48 hpi, the number of DEGs was 153 (107 were upregulated and 46 were downregulated). At 96 hpi, the number of DEGs significantly increased to 666 (207 upregulated and 459 downregulated genes) (Figure 5A,B). A Venn diagram showed a comparison of DEGs (differentially expressed genes) from the two comparisons of different treatments at the same time points. Only 38 common DEGs were identified in the comparisons (Figure 5C). These results indicate that white light impaired mycoparasitic and infection activities of *T. roseum* by modulating gene expression in a stage‐dependent manner. To further identify the biological functions of the DEGs at 48 and 96 hpi, KEGG enrichment analyses were performed. Notably, the genes in HOG‐MAPK, tryptophan metabolism, and tyrosin metabolism pathways were significantly enriched (Figure S1), suggesting that the genes associated with these pathways may play crucial roles in the mycoparasitic and infection activities. These results demonstrate that white light modulated developmental processes of *T. roseum* via the regulation of different pathways.

### 3.5 White Light‐Orchestrated Expression of *T. roseum* Effectors

**FIGURE 6:**
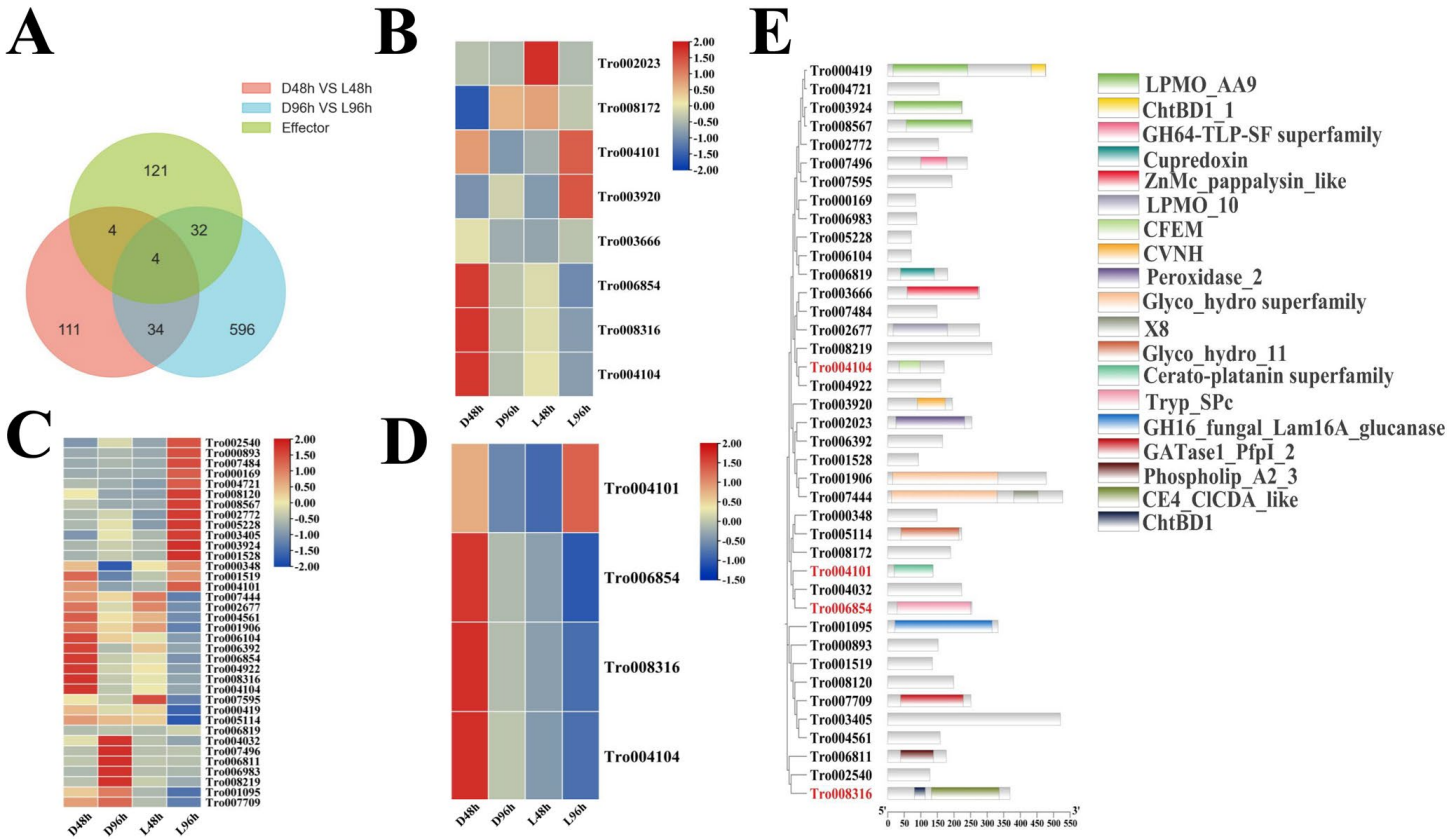
Expression of effectors in *T. roseum* in darkness or under white light condition at 48 and 96 hpi. (A) A Venn diagram of common genes in effectors and DEGs at 48 and 96 hpi. The expression of common effectors at 48 hpi (B) and 96 hpi (C). (D) The expression of four common effectors. (E) Phylogenetic and domain analysis of differentially expressed effectors. The four common effectors were highlighted in red. Each expression value at different time points represented the average of normalized FPKM of three biological replications.

To analyze the white light‐modulated expression of effector genes in *T. roseum* , the effectors were screened and analyzed. A total of 161 effectors were predicted in the *T. roseum* genome (Table S1). Although the expression of the 161 effectors was detectable and showed different expression patterns at 48 and 96 hpi (Figure S2A,B), 8 and 36 effectors were respectively DEGs at 48 and 96 dpi (Figure 6A). At 48 hpi, three effector genes were upregulated and five were downregulated. At 96 hpi, 16 effector genes were upregulated and 20 were downregulated. There were four common differentially expressed effectors with significantly downregulated patterns at 48 or 96 hpi, indicating white light may downregulate the core effectors to impair mycoparasitic and infection processes of *T. roseum* (Figure 6D). The differentially expressed effectors contained several domains, such as CFEM, CVNH, and peroxidase (Figure 6E).

### 3.6 White Light‐Mediated Expression Patterns of the Genes in HOG‐MAPK, Tryptophan Metabolism, and Tyrosine Metabolism Pathways

**FIGURE 7:**
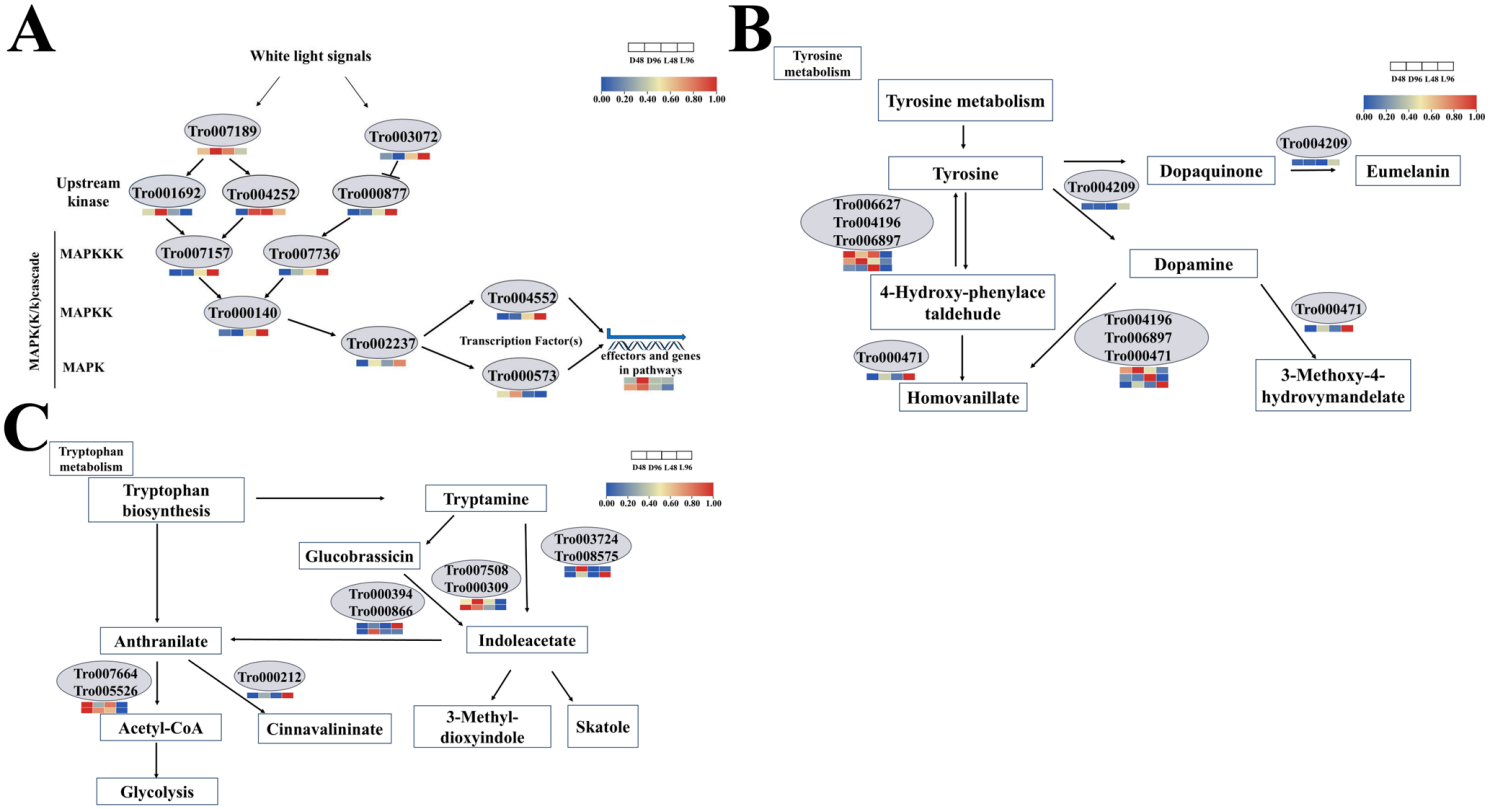
Expression of DEGs in HOG‐MAPK (A), tryptophan metabolism (B), and tyrosine metabolism (C) pathways. Each expression value of genes at different time points represented the average of normalized FPKM of three biological replications.

Since the HOG‐MAPK, tryptophan metabolism, and tyrosine metabolism pathways were shown to be crucial for fungal development and these pathways were identified in the KEGG pathways of DEGs (Figure S1), gene expression in these pathways was analyzed (Figure 7 and Table S2). In the HOG‐MAPK pathway, Cla4 and Ste20 were significantly downregulated by white light; however, Pbs2 and Ypd1 were notably upregulated. In the tyrosine metabolism pathway, two DEGs (Tro006627 and Tro004196) were downregulated and three DEGs (Tro006897, Tro004209, and Tro000471) were upregulated. In the tryptophan metabolism, five DEGs (Tro007664, Tro005526, Tro000212, Tro007508, and Tro000309) were downregulated and four DEGs (Tro000212, Tro000394, Tro000866, and Tro008575) were upregulated.

### 3.7 White Light‐Mediated Expression of Light Receptor and Validation of Gene Expression in RNA‐Seq by RT‐qPCR

**FIGURE 8:**
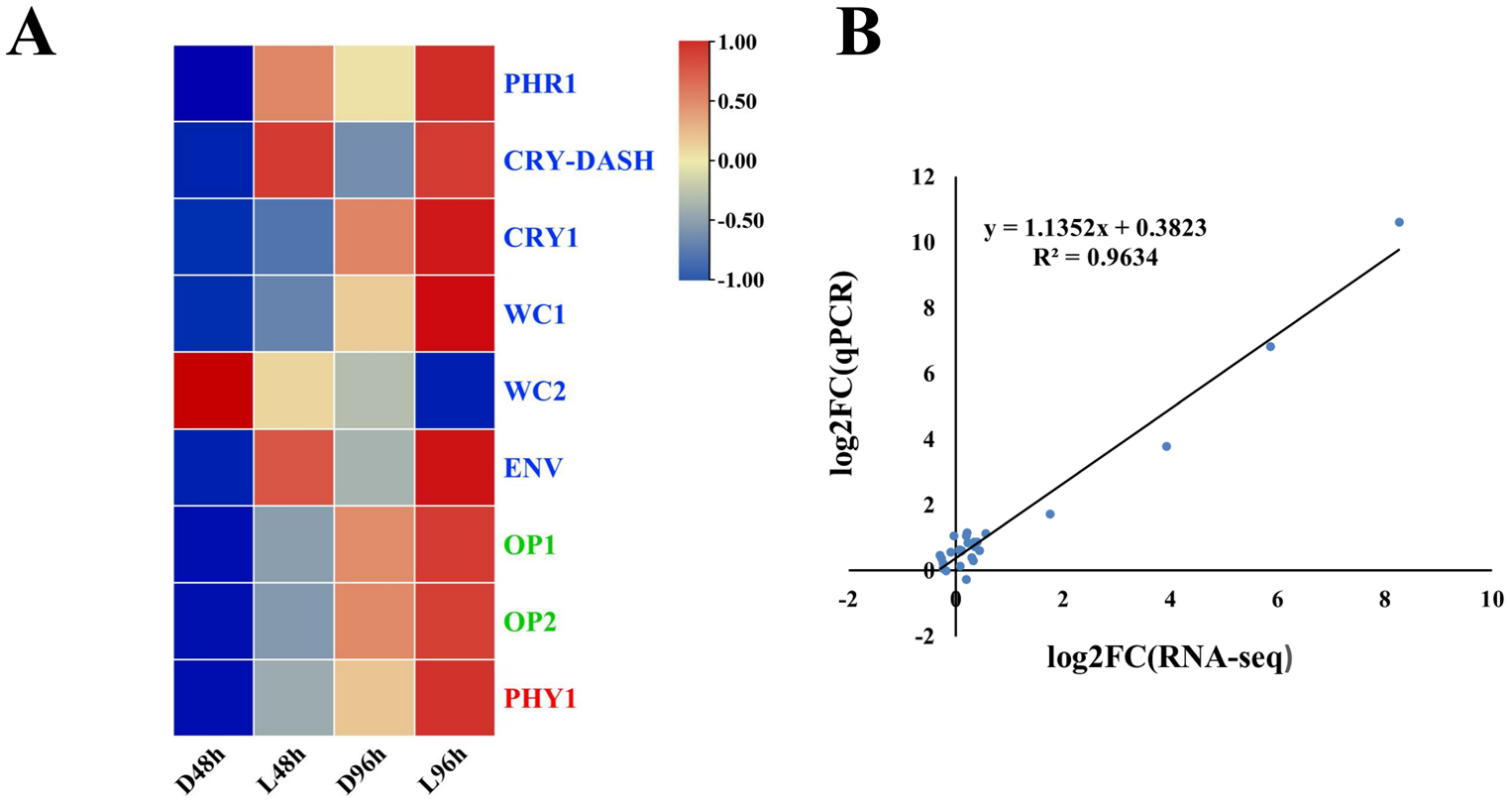
(A) Expression of nine light receptors in *T. roseum* in darkness and under white light condition at different time points. (B) Linear regression analysis of relationships between RNA‐seq and RT‐qPCR results. Each expression value of genes in transcriptome at different time points represented the average of normalized FPKM of three biological replications. The gene expression levels in RT‐qPCR were calculated using the $2^{-\Delta \Delta C_{T}}$ method with three biological replications.

To characterize the expression patterns of light receptors in darkness and under white light condition, the receptors were screened (Table S3). In total, nine light receptors, including six blue light receptors, two green light receptors, and one red light receptor, were found (Figure 8A). Compared to those in darkness, the expression of blue light receptors CRY‐DASH, PHOTOLYASE (PHR), and ENVY (ENV), green light receptors OPSIN 1 (OP 1) and OPSIN 2 (OP 2) was significantly upregulated under white light condition (Figure 8A). To validate the DEGs identified by the RNA‐seq analysis, the expression of four light receptors and nine genes in the HOG‐MAPK pathway (Table S2) was analyzed by RT‐qPCR. The RT‐qPCR results showed that the expression patterns of the selected genes were highly consistent with those obtained by RNA‐seq analysis, suggesting the transcriptome results were validated (Figure 8B).

## 4 Discussion

### 4.1 White Light Impairs Mycoparasitic and Infection Activities of *T. roseum*

It has been repeatedly shown that light is capable of modulating developmental and metabolic processes of fungi (Schumacher 2017; Yu and Fischer 2019; Moreno‐Ruiz et al. 2020; Bayram and Bayram 2023; Sun et al. 2024). However, little is known about the effects of light on *T. roseum*, an agriculturally and economically important fungus infecting plants and mycoparasiting on phytopathogens (Zhu, Duan, Cai, Zhang, et al. 2022; Zhang et al. 2024; Zhao et al. 2024). It was found that white light impaired infection on tomato fruit and mycoparasitic activities on the wheat powdery mildew fungus, *B. graminis* f. sp. *tritici* (Figures 1 and 2). Additionally, white light inhibited colony expansion and triggered conidiation in vitro (Figure 3). These results were consistent with previous studies illustrating that white light modulated conidiation, infection, and mycoparasitism of fungi (Karlsson et al. 2017; Yu and Fischer 2019; Moreno‐Ruiz et al. 2020; Speckbacher et al. 2020; Bayram and Bayram 2023; Sun et al. 2024). It was demonstrated that red and far‐red light, which is sensed by red receptor phytochrome FPH1, increased the mycoparasitic activities of *Trichoderma guizhouense* on *Alternaria alternata* and *Fusarium oxysporum* (Sun et al. 2024). However, in this study, the mycoparasitic activities of *T. roseum* were decreased under white light conditions. The differences might be caused by the sensing of blue and green light via blue and green light receptors, which negatively regulated the infection and mycoparasitic activities of *T. roseum* . Whether and how blue and green light receptors modulate mycoparasitic activities remain unclear. More research is needed to study the effects and molecular mechanisms of blue and green light on the mycoparasitic activities of *T. roseum* .

### 4.2 White Light Orchestrates the Expression of Effectors in *T. roseum*

It is repeatedly illustrated that effectors are crucial for phytopathogens to suppress the plant host resistance response and for enhancing the biocontrol efficiency of mycoparasites on plant diseases (Laur et al. 2018; Kusch et al. 2023; Santhanam et al. 2023; Seong and Krasileva 2023; Bilstein‐Schloemer et al. 2025). Although the genome assembly of *T. roseum* is available, little is demonstrated about the effectors of this fungus and thus the highly complex *T. roseum‐*host interactions are obscure (Zhu et al. 2023). By genome‐wide screening, 161 effectors were obtained and their expression in darkness and under white light conditions was analyzed (Figure S2 and Figure 6A). Eight (three upregulated and five downregulated) effectors were differentially expressed at 48 hpi and the number increased to 36 (16 upregulated and 20 downregulated) at 96 hpi (Figure 6B,C). Four common differentially expressed effectors were notably downregulated by white light (Figure 6D), suggesting white light impaired mycoparasitic and infection activities by downregulated effectors in *T. roseum*. However, the functions of these four effectors remain unknown. Further research is required to characterize the functions of these effectors.

### 4.3 White Light Modulates the Expression of Genes in Different Pathways and Light Receptors

Although the genome assembly of *T. roseum* is available, little is known about the genes related to white light‐mediated developments of this fungus (Zhu et al. 2023). Tryptophan metabolism, tyrosine metabolism, and HOG‐MAPK pathway were shown to play crucial roles in fungal developmental processes (Yang et al. 2020; Zhang et al. 2021; Cui et al. 2022; Cruz‐Mireles et al. 2024; Wang et al. 2024). In coherence with previous studies, the expression of genes in the HOG‐MAPK, tryptophan metabolism, and tyrosine metabolism pathways was notably modulated by white light during developments (Figure 7). These results indicate that white light regulates the expression of genes in the different pathways of *T. roseum* and that tryptophan and tyrosine may be involved in conidiation, mycoparasitic, and infection activities.

Fungi sense white light with light receptors. In this study, three blue light receptors (CRY‐DASH, PHOTOLYASE, and ENVY) and two green light receptors (OPSIN 1 and OPSIN 2) were significantly upregulated under white light conditions, indicating these light receptors may be involved in white light‐mediated mycoparasitic and infection activities in *T. roseum*. The red light receptor phytochrome was speculated to be associated with the mycoparasitic activities of *T. guizhouense* (Sun et al. 2024). However, in our study, the expression of the red light receptor was slightly upregulated by white light. Future studies should be conducted illustrating whether the red light receptors were capable of orchestrating fungal developments in darkness and/or whether the red light receptor‐mediated mycoparasitic activities were in a fungal species‐dependent manner. Although blue light receptors were repeatedly shown to play roles in fungal developments, the functions of green light receptors (opsins) in fungi were rarely studied (Yu and Fischer 2019; Bayram and Bayram 2023). An opsin binds to retinal to form a rhodopsin that provides light‐dependent ion transport and sensory functions (Ding et al. 2024). In our study, white light significantly upregulated the expression of opsins and the genes required for the chromophore (retinal) synthesis (Figure 8 and Figure S1), indicating the opsins and retinal might be crucial for fungal developments. However, the exact functions of fungal opsins and retinal were not clear. More research is needed illustrating the functions of opsins and retinal in fungi.

## 5 Conclusions

These findings provide novel insights into the molecular mechanism of white light‐regulated mycoparasitic and infection activities of *T. roseum*, the causal agent of postharvest pink rot and a mycoparasitism of fungi and insects. Our results indicate the following: (1) white light impairs mycoparasitism and infection processes of *T. roseum* by regulating gene expression, highlighting the significance of the light signal for the development of *T. roseum* ; (2) the expression of effectors, light receptors, and genes in tryptophan metabolism, tyrosine metabolism, and HOG‐MAPK pathways was orchestrated by white light in a developmental stage‐dependent manner; and (3) the discovered genes that are related to the mycoparasitic and infection processes of *T. roseum* will be targets for function characterization in future studies.

## Author Contributions

**Mo Zhu:** conceptualization (equal), funding acquisition (equal), methodology (equal), supervision (equal), writing – original draft (equal). **Fuhai Zhang:** data curation (equal), investigation (equal), visualization (equal). **Zongbo Qiu:** funding acquisition (equal), supervision (equal). **Sujing Zhao:** supervision (equal), visualization (equal). **Shiqiang Gao:** methodology (equal), writing – review and editing (equal).

## Ethics Statement

The authors have nothing to report.

## Consent

The authors have nothing to report.

## Conflicts of Interest

The authors declare no conflicts of interest.

## Supporting information


**Figure S1.** GO terms and KEGG enrichment of DEGs. Top 30 GO terms of DEGs at 48 hpi (A) and 96 hpi (B). Top 20 KEGG enrichment of DEGs at 48 hpi (C) and 96 hpi (D).
**Figure S2.** Effectors and their expression patterns at 48 and 96 hpi. (A) A Venn diagram of shared effectors, expressed genes, and DEGs. (B) Expressions of identified effectors at 48 and 96 hpi under white light (L) and in darkness (D) conditions.

**Table S1.** Expression value and annotation of the identified effectors.

**Table S2.** Expression value and annotation of the identified genes in different pathways.

**Table S3.** Primers of genes for RT‐qPCR assays.

## Data Availability

Raw sequencing reads of transcriptome data were deposited in the National Center for Biotechnology Information database under accession number PRJNA1110597 and are available at the following URL: https://www.ncbi.nlm.nih.gov/bioproject/PRJNA1110597/.
