# Supplementary figures and images for: White Light Orchestrates Mycoparasitic and Infection Activities by Regulating Expression of Effectors in Trichothecium roseum
Source: Food Sci Nutr. 2025 Jun 28;13(7):e70396. doi: 10.1002/fsn3.70396 (PMC12205212; doi:10.1002/fsn3.70396)

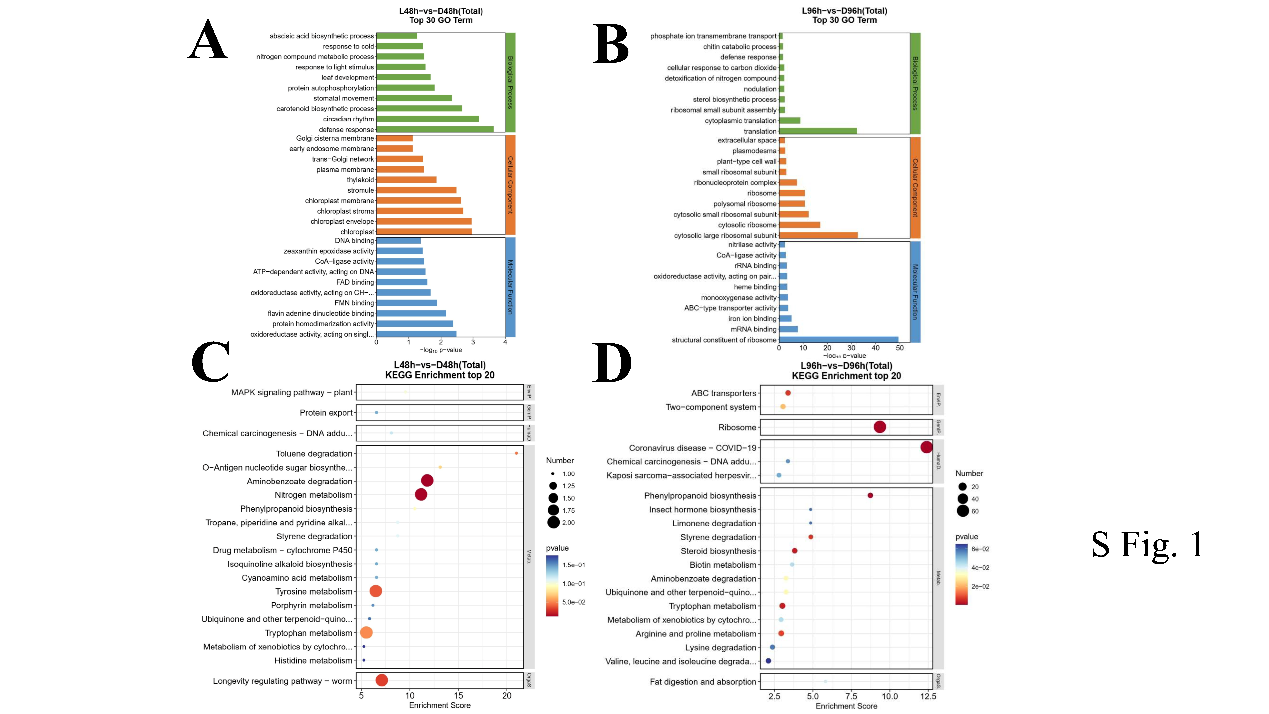


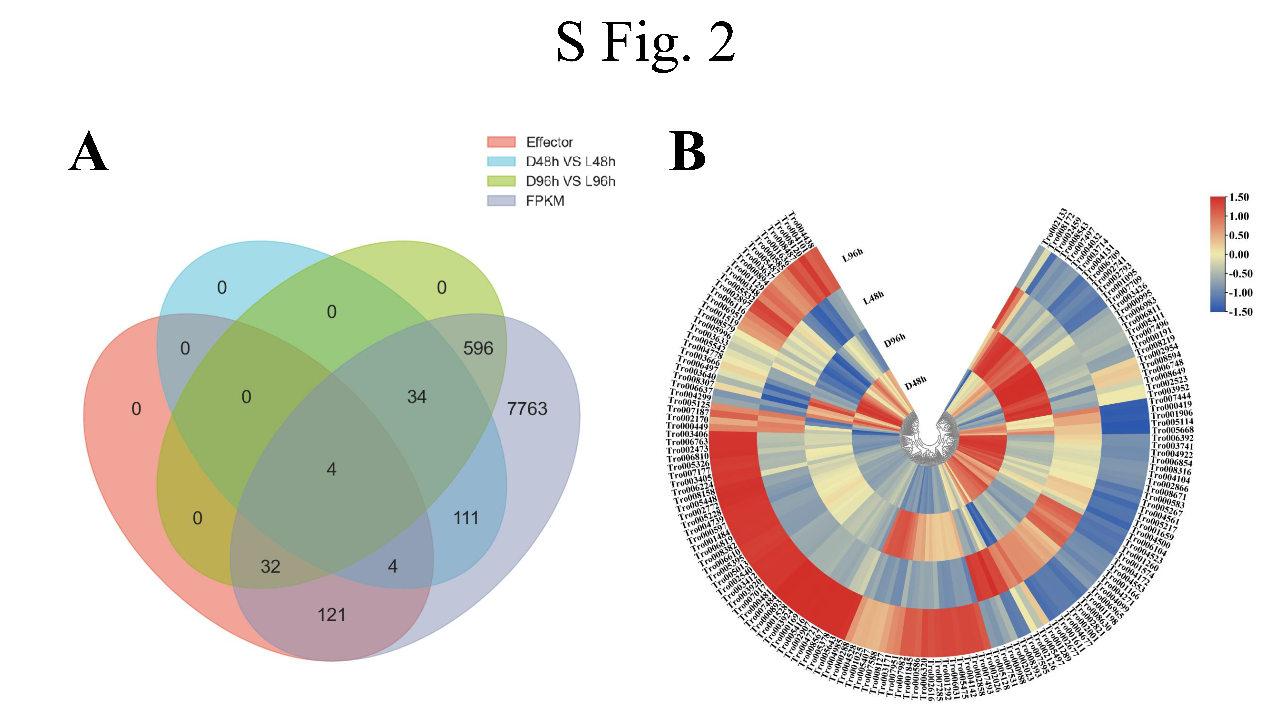

Supplement: Supplementary file 1 — Figure S1. GO terms and KEGG enrichment of DEGs. Top 30 GO terms of DEGs at 48 hpi (A) and 96 hpi (B). Top 20 KEGG enrichment of DEGs at 48 hpi (C) and 96 hpi (D). Figure S2. Effectors and their expression patterns at 48 and 96 hpi. (A) A Venn diagram of shared effectors, expressed genes, and DEGs. (B) Expressions of identified effectors at 48 and 96 hpi under white light (L) and in darkness (D) conditions. [file FSN3-13-e70396-s002.docx]
